# A cross-sectional study of herbal medicine use and contributing factors among pregnant women on antenatal care follow-up at Dessie Referral Hospital, Northeast Ethiopia

**DOI:** 10.1186/s12906-022-03628-8

**Published:** 2022-05-24

**Authors:** Yaschilal Muche Belayneh, Tewodros Yoseph, Solomon Ahmed

**Affiliations:** Department of Pharmacy, College of medicine and Health Sciences, Wollo University, P.O. Box 1145, Dessie, Ethiopia

**Keywords:** Pregnant women, Herbal medicine, Current pregnancy, Associating factors

## Abstract

**Background:**

Herbal medicines are widely used in the world especially in developing countries. Pregnant women use herbal products to treat pregnancy related illnesses due to prior experience of herbal medicine use and easy accessibility of the products with less cost. However, herbal products could affect fetal growth and contribute to maternal and fetal morbidity and mortality. Herbal drug use during pregnancy is not well studied in Ethiopia specifically in northeast Ethiopia.

**Methods:**

A cross-sectional survey was conducted among 254 pregnant women on antenatal care follow-up at Dessie referral hospital. Semi-structured questionnaires were used for data collection. After collection, data were coded, entered and analyzed by SPSS version 20. Chi squared test and Logistic regression were used to evaluate the association between dependent and independent variables.

**Result:**

Among the total of 254 respondents, 130 (51.2%) used herbal drugs during current pregnancy. The most commonly mentioned reason for herbal drug use was “herbal medicines are accessible without prescription” (43.1%). The herbal medicines used were Ginger (*Zingiber officinale* Roscoe) (43.8%), followed by Garlic (*Allium sativum* L.) (23.8%), Damakese (*Ocimum lamiifolium* Hochst. ex Benth.) (21.5%) and Tena-adam (*Ruta chalepensis* L.) (10.8%). The indications for herbal drug use were nausea/vomiting (43.8%), headache (30.8%) and common cold (25.4%). The most commonly mentioned sources of information on herbal medicine were families and friends (80.0%) followed by neighbors (12.3%), and the most commonly cited sources of herbal products were market (67.7%) and self-preparation (20.0%). Being illiterate or having only primary school education (Adjusted Odds Ratio [AOR]: 3.717, 95% CI: 0.992-13.928), having secondary school education background (AOR: 3.645, 95% CI: 1.394-9.534), and poor monthly income (AOR: 7.234, 95% CI: 2.192-23.877) were the variables that showed significant association with herbal drug use during current pregnancy.

**Conclusion:**

This study showed that half of the sampled pregnant women used herbal medicine during current pregnancy, and education status and monthly income level of the women were associated with herbal drug use.

**Supplementary Information:**

The online version contains supplementary material available at 10.1186/s12906-022-03628-8.

## Background

World health organization revealed that about 80% of the population in developing countries rely on nonconventional medicine mainly of herbal sources in their primary healthcare [1]. Pregnancy is associated with different physiological changes resulting in various ailments. These pregnancy-related ailments often cause pregnant women to start self-medication including the use of herbal products [2]. However, research gaps and lack of regulatory framework on herbal medicines across sub-Saharan Africa are critical problems as herbal drug use during pregnancy raises several concerns of safety [3].

Reports have estimated that around 80% of the population in Africa use traditional medicines and about 85% of traditional medicine involves use of herbal preparations [4]. In Ethiopia, 80% of the population use traditional medicine [5], and more than 95% of traditional medicinal preparations are herbal products [6]. The wide use of herbal drugs may be due to easy accessibility, affordability and acceptability of folk medicines by the population in developing countries [7]. Pregnant women are one of those populations exposed to herbal medicine use due to pregnancy induced changes in normal physiology and pregnancy related ailments [5]. However, the use of herbal products during pregnancy has safety concerns. Exposure of pregnant women to herbal chemicals during pregnancy could affect maternal health and fetal development [8, 9]. For instance, a preliminary study in rats revealed that prenatal exposure to high dose of ginger results in increased fetal loss, increased fetal weight and bone maturation [10]. Though human studies are limited due to ethical reasons, some epidemiological studies on pregnant women indicated that herbal use could influence pregnancy outcomes including risk of abortion or preterm labor, presence of malformations, intrauterine growth and neonatal birth weight [11]. A study revealed that the use of *Rhizoma coptidis* for skin conditions during first trimester of pregnancy causes nervous system and external genital organ malformations in the offspring [12]. Moreover, use of *Angelica sinensis* and *Petroselinum crispum* for prevention of miscarriages during the first trimester was associated with connective tissue, eye and musculoskeletal congenital anomalies in the offspring [12]. Additional concerns including poor regulatory framework for manufacturing, importation and distribution, adulteration and lack of standardization of herbal preparations in Africa as well as herb-drug interactions make herbal medicine use risky for pregnant women [3, 13]. Similarly, available registered herbal products does not comply with good manufacturing practices as well as safety and efficacy principles as is required for conventional medicines [2, 14].

Advanced investigations on the potential benefits and risks of herbal product use during pregnancy require baseline data on the magnitude of herbal drug use during pregnancy and type of medicinal plants used. Nevertheless, data available on herbal drug use during pregnancy in Africa including Ethiopia is scarce. Herbal drug use including the specific medicinal plants used among pregnant women is not yet studied specifically in the northeast region of Ethiopia. As a result, this study was conducted to assess herbal medicine use and contributing factors among pregnant women on antenatal care follow-up at Dessie referral hospital.

## Methods

### Study area and period

The study was conducted at Dessie referral hospital, South Wollo zone in the Amhara region of Ethiopia. Dessie is a town of South Wollo zone located at 401 km from Addis Ababa. The total area of the town is about 15.08 km^2^ with a population of more than 610,000. In the town, there is one referral hospital which is the biggest service delivering referral hospital to the dweller of the town and surrounding community. The referral hospital has different departments and wards like outpatient department, surgical ward, medical ward and the emergency ward including the emergency service and follow up of chronic diseases like TB, DM and HIV AIDS. It also has antenatal care (ANC) department which provide services that can prevent, detect and treat risk factors early in the pregnancy. Data collection was conducted for one month from May 6 to June 5, 2019 at the ANC department of the hospital.

### Study Participants

#### Study design

A cross sectional survey was carried out among pregnant women on ANC follow-up at DRH using semi-structured questionnaires.

Source and study population of the study: All pregnant women attending DRH were considered as the source population, and all pregnant women on antenatal care follow up at DRH during the data collection period (from May 6 to June 5, 2019) were considered as study population for the study.

#### Inclusion and Exclusion criteria

The inclusion criterion was being a pregnant woman with age greater than or equal to18 years. Pregnant women who were unable to hear or communicate and mentally disabled women were excluded from the study.

### Sample size determination and sampling technique

Prevalence of herbal drug use during pregnancy at Hossana town, 73.1% [15], 95% confidence interval and 5% margin of error were used to calculate the sample size using the single proportion formula. Accordingly, the sample size was calculated to be 302. Since the total number of pregnant women attending ANC department of DRH was 1200, which is less than 10,000, reduction formula was used and the sample size was reduced to 242. Then, 5% contingency was added for possible nonresponse and the final sample size was calculated to be 254 pregnant women. Pregnant women attending the ANC clinic during the one month data collection period were included in the study. Thus, convenience sampling was the sampling technique employed in the study.

### Study variables

Herbal medicine use is the dependent variable of this study. Age, Marital status, Number of children, Occupation, Monthly income, residence, Distance from health facilities, Stage of pregnancy and gravida are the independent variables that can possibly affect the dependent variable, herbal medicine use.

### Data collection tool and procedure

Data were collected by using semi-structured interviewer administered questionnaire (Additional file 1). The data collection tool (questionnaire) was first prepared in English language based on the aim of the study using a questionnaire of previous similar study as a guide [16]. The questionnaire was then translated to Amharic language. The questionnaire has three sections; section I (Socio-Demographic Information), section II (Obstetrics Information) and section III (Herbal Medicine Use). Pretested data collection questionnaire papers were used for data collection. The aim of the research was mentioned to each study participant and informed consent was received before the data collection. Data were collected by graduating class pharmacy students of Wollo University. The list of local (Amharic) names of medicinal plants or herbs mentioned by the informants was submitted to the Biology department, Wollo University for authentication of the scientific name by the botanist.

### Data quality control, processing and analysis

After the questionnaire is prepared, pretest was done for validity and reliability of the questionnaire. Content validity was used to evaluate the validity of the data collection tool. Cronbach’s alpha test was used to measure the reliability, and cronbach’s alpha coefficient was calculated to be >0.70. The data were cleared and checked every day for consistency and completeness.

After completing data collection, the collected data were coded, entered and analyzed using SPSS version 20. Chi squared test and Logistic regression (binary and multivariate) were used to evaluate the association between independent variables (associated factors) and herbal medicine use using COD and AOD at 95% confidence interval, and a P-value <0.05 was considered statistically significant. Variables that showed significant (p<0.05) association with herbal medicine use in the chi squared test were selected for binary logistic regression analysis. Then, variables with P value less than 0.25 in the binary logistic regression analysis were included in the multivariate analysis. Study variables were summarized in tables using frequencies and percentages.

### Ethical consideration

Permission to conduct the study in DRH was requested with a formal letter from Wollo University. Ethical approval was received from the ethical review committee of college of medicine and health sciences, Wollo University (reference number, WU/1224/08/11) before data collection. Verbal informed consent was received from each study participant after clarifying the objective of the study. Confidentiality of the information obtained was ensured throughout the study.

## Result

### Socio-Demographic information of the study participants

A total of 254 pregnant women were included in this study with 100% response rate. Out of 254 pregnant women who participated in this study, 74% were in the 20-30 years of age group and most (97%) were married. Considering their occupation, 82 (32.3%) were self-employed and 81 (31.9%) were house wife. With regard to monthly income, 48.4% had a low income (1,381 to 6,900 Ethiopian birr). Concerning educational level; 49 (19.3%) of the respondents have primary education and 98(38.6%) respondents have secondary education background. Majority of respondents 251 (98.8%) were Amhara, and 138(54.3%) respondents were Muslims and 111(43.2%) were orthodox Christianity followers (Table 1).

**Table 1:** Socio-demographic information of the respondents

Variables	Categories	Frequency (N)	Percentage (%)
Age in years	Less than 20 years	5	2.0
	21-30 years	188	74.0
	>30 years	61	24.0
Marital status	Single	1	4.0
	Married	248	97.6
	Widowed	5	2.0
Occupation status	Governmental employed	68	26.8
	Self-employed	82	32.3
	House wife	81	31.9
	Farmer	14	5.5
	Student	9	3.5
Monthly income (ETB)	<1,380 (poor)	72	28.3
	1,381–6,900 (low)	123	48.4
	6,901–13,800 (middle)	59	23.2
Educational level	Illiterate	8	3.1
	Primary school	49	19.3
	Secondary school	98	38.6
	Diploma/degree	99	39.0
Ethnicity	Amhara Tigre	251 3	98.8 1.2
Religion	Orthodox	111	43.7
	Muslim	138	54.3
	Protestant	5	2.0
Place of residence	Urban Rural	224 29	88.2 11.4
Distance from health facility	<5 km	202	79.5
	5-10 km	16	6.3
	>10 km	36	14.2

ETB, Ethiopian Birr

### Obstetric characteristics of the study participants

Among the 254 pregnant women interviewed 132 (52.0%) were gravida two and 67 (26.4%) were gravida one. Among the total of 187 respondents with gravida 2 and above, 140 (55.1%) had 1 child and 20 (7.9%) had abortion history, of which 10 (50.0%) were due to known illnesses and 9 (45%) were due to unknown causes (Table 2).

**Table 2:** Obstetric characteristics of the respondents

Variables	Categories	Frequency (N)	Percentage (%)
Number of gravid	One	67	26.4
	Two	132	52.0
	Three	42	16.5
	Four and above	13	5.1
Number of children	No child	70	27.6
	One child	140	55.1
	Two children	36	14.2
	Three and above	8	3.1
Previous abortion	Yes No	20 234	7.9 92.1
Reason for abortion	Unknown illness	9	45.0
	Known illness	10	50.0
	Unwanted pregnancy	1	5.0
Stage of pregnancy	First trimester	4	1.6
	Second trimester	156	61.4
	Third trimester	94	37.0

### Prevalence of herbal medicine use among the respondents

Of the total respondents, 130 (51.2%) used herbal drugs during their current pregnancy, and 78 (30.9%) of them used during their second trimester. Additionally, 103 (40.6%) used herbal drug during their previous pregnancy (Table 3).

**Table 3:** Prevalence of herbal medicine use among the respondents

Variables	Categories	Frequency (N)	Percentage (%)
Prevalence of herbal medicine use	During current pregnancy During prior pregnancies	130 103	51.2 40.6

### Indications of herbal products and the name of medicinal herbs used among respondents

The herbal products used were Ginger (*Zingiber officinale* Roscoe) (43.8%), followed by Garlic (*Allium sativum* L.) (23.8%), Damakese (*Ocimum lamiifolium* Hochst. ex Benth.) (21.5%) and Tena-adam (*Ruta chalepensis* L.) (10.8%). The reported indications for the herbal medicines were nausea/vomiting (43.8%), headache (30.8%) and common cold (25.4%) as presented in Table 4.

**Table 4:** Indications of herbal products and the name of medicinal herbs used

Variables		Frequency (N)	Percentage (%)
Indications	Nausea/vomiting	57	43.8
	Headache	40	30.8
	Common cold	33	25.4
Name of herbs	Ginger (*Zingiber officinale* Roscoe)	57	43.8
	Garlic (*Allium sativum* L.)	31	23.8
	Damakese (*Ocimum lamiifolium* Hochst. ex Benth.)	28	21.5
	Tena-adam (*Ruta chalepensis* L.)	14	10.8

### Source of information on herbal medicine and source of herbal products

The most commonly cited sources of information were families and friends (80.0%) followed by neighbors (12.3%), and the most common sources of herbs were market place (67.7%), followed by self-preparation (20.0%) (Table 5).

**Table 5:** Source of information on herbal medicine and source of the herbal preparations

Variables	Categories	Frequency (N)	Percentage (%)
Source of information	Traditional healers	9	6.9
	Family and friends	104	80.0
	Neighbors	16	12.3
	Other	1	0.8
Source of herbs	Self-preparation	26	20.0
	Market place	88	67.7
	Neighbors	16	12.3

### Reasons mentioned for herbal drug use

The most commonly mentioned reason for using herbal medicines was “herbal medicines are accessible without prescription” (43.1%), followed by “herbal medicines are effective than conventional medicines” (28.1%) (Table 6).

**Table 6:** Reasons mentioned by the respondents for herbal medicine use

Reasons for herbal medicine use	Frequency (N)	Percentage (%)
Herbal medicines are accessible without prescription	109	43.1
Herbal medicines are effective than conventional medicines	71	28.1
Herbal medicines have lower cost	53	20.9
Herbal medicines are safe during pregnancy	20	7.9

### Reasons mentioned for not using herbal drugs

The most commonly mentioned reason for not using herbal medicine was “didn’t get sick during gestation” (46.3%), followed by “fear of the side effects” (26.8%) (Table 7).

**Table 7:** Reasons mentioned by the respondents for not using herbal medicine

Reasons for not using herbal medicine	Frequency (N)	Percentage (%)
Didn’t get sick during gestation	19	46.3
Fear of side effects	11	26.8
Lack of belief in the benefit of herbs	10	24.4
Unavailability of herbal medications	1	2.5

All the herbal medicine users reported no undesirable response was experienced after the use of herbal products. Unfortunately, only 12.3% of the women responded that they were satisfied with the efficacy of the herbal products used.

### Factors contributing to herbal medicine use

Independent variables that showed significant association with herbal drug use in the Chi Squared test and bivariate logistic analysis were further analyzed in multivariate regression. Finally, education level and average monthly income showed significant association (P < 0.05) with the dependent variable in multivariate regression analysis (Table 8). The odds of herbal drug use during current pregnancy was 3.717 times higher among illiterate and women with primary school education as compared to respondents with college diploma/degree (AOR: 3.717, 95% CI: 0.992-13.928), and the odds of herbal medicine use was 3.645 times higher among pregnant women with secondary school education as compared to women who have college diploma/degree (AOR: 3.645, 95% CI: 1.394-9.534). Similarly, women who were poor (monthly income less than 1,380 birr) were 7.234 times more likely to use herbal products than those who had middle monthly income of 6,901-13,000 Ethiopian birr (AOR: 7.234, 95% CI: 2.192-23.877). There was no statistically significant association (P > 0.05) between herbal medication use and occupation, place of residence, distance from health institution, number of gravid and number of children in multivariate logistic analysis.

**Table 8:** Factors associated with herbal medicne use during pregnancy

Variables	Categories	Chi square	Herbal medicine use		P value	COR(95%CI)	*P* value	AOR(95% CI)
			No	Yes				
Occupation	Governmental Employee	0.001	55	13	0.001	0.152 (0.054-0.427)	0.360	0.487 (0.104-2.271)
	Self-employed		49	33	0.083	0.433 (0.168-1.116)	0.915	1.084 (0.249-4.724)
	Housewife		9	14	0.009	4.091 (1.430-11.706)	0.153	3.165 (0.653-15.348)
	Others*		11	70		1.00		1.00
Monthly income	<1,380 (poor)	0.001	45	14	0.001	29.847 (11.161-79.819)	0.001	7.234 (2.192-23.877)
	1381–6900 (low)		72	51	0.021	2.277 (1.132-4.580)	0.216	1.708 (0.731-3.987)
	6901–13800 (middle)		7	65		1.00		1.00
Education level	Illiterate/primary school	0.001	8	49	0.001	24.194 (9.895-59.152)	0.05	3.717 (0.992-13.928)
	Secondary school		37	61	0.001	6.512 (3.439-12.330)	0.008	3.645 (1.394-9.534)
	Diploma/Degree		79	20		1.00		1.00
Residence	Urban	0.001	121	103	0.001	10.573 (3.117-35.862)	0.972	1.049 (0.070-15.648)
	Rural		3	27		1.00		1.00
Distance from health facility	<5km	0.001	113	89	0.001	0.098 (0.034-0.289)	0.191	0.198 (0.018-2.241)
	5-10km		7	9	0.012	0.161 (0.038-0.674)	0.265	0.230 (0.017-3.050)
	>10km		4	32		1.00		1.00
Number of gravid	One	0.001	41	26	0.001	0.217 (0.099-0.473)	0.914	0.827 (0.026-26.206)
	Two		69	63	0.001	0.312 (0.155-0.625)	0.402	0.363 (0.034-3.890)
	Three and above		14	41		1.00		1.00
Number of child	No child	0.001	43	27	0.001	0.263 (0.118-0.590)	0.431	0.243 (0.007-8.256)
	One child		68	72	0.029	0.444 (0.215-0.919)	0.694	0.600 (0.047-7.637)
	Two and above		13	31		1.00		1.00

*Farmer and student; *COR* Crude Odds Ratio, *AOR* Adjusted Odds Ratio, *CI* Confidence Interval

## Discussion

The findings of this study showed that 51.2% of the pregnant women at Dessie referral hospital, northeast Ethiopia used herbal medicine during their current pregnancy. This is almost equal to the prevalence reported in a study done at University of Gondar teaching hospital (northwest Ethiopia), 48.6% [16] and Nekemte hospital (west Ethiopia), 50.4% [5]. Additionally, prevalence of herbal drug use reported in the current study is within the range of 22.3 to 82.3% which was reported in a review of published literatures from Middle East countries [2]. In the contrary, prevalence of herbal drug use in the current study is lower compared to a study done at health facilities of Hosanna Town, Southern Ethiopia (73.1%) [15] and higher than the study done in Imo state of Nigeria (36.8%) [17]. The possible explanation for this might be differences in accessibility and affordability of herbal products and cultural differences among study participants.

This study showed easy accessibility, effectiveness, lower cost and fewer side effects of herbal medicines were the reasons mentioned by the respondents for using herbal drugs. This finding is in line with other studies carried out at Nekemte Hospital [5], health facilities of Hossana town [15] and a review of published literatures from Middle East countries [2] indicating similar reasoning for herbal drug use.

The current study revealed that *Zingiber officinale, Allium sativum*, *Ocimum lamiifolium* and *Ruta chalepensis* were the medicinal herbs used during pregnancy. This finding is comparable to the results of other studies conducted at Harar hosptals [18], Nekemte hospital [5], and health facilities of Hossana town [15] in Ethiopia and a tertiary Hospital in Northern Nigeria [19]. This implies similarity of herbal medicines utilized by pregnant women in different parts of Ethiopia as well as Africa.

According to this study, herbal drugs were used to treat nausea/vomiting, headache and common cold. The reasons mentioned for using herbal products were “herbal medicines are accessible without prescription, more effective than conventional medicines, have lower cost and safe during pregnancy”.

A randomized controlled trial which was done among pregnant women with symptoms of nausea and vomiting showed consumption of 1.5g of dried ginger for 4 days improves nausea and vomiting without affecting birth outcomes [20]. An experimental study on rats suggests the antiemetic effect of ginger may be largely due to its antagonistic effect on 5-HT_3_ receptor [21]. Similarly, a review of experimental and clinical studies showed *Allium sativum* L. has antioxidant, anti-inflammatory, antibacterial, antihypertensive, antidyslipidemic and anticancer activities [22]. However, experimental studies on animals showed embryotoxic effects of *Ruta chalepensis* [23] and *Zingiber officinale* [24, 25]. Additionally, pregnant women should avoid the use of garlic (*Allium sativum*) prior to surgery including caesarean section due to its anti-hemostatic effect which can lead to excessive bleeding [22, 26]. There is no pregnancy related safety data on *Ocimum lamiifolium.*

This study showed poor monthly income is significantly associated with herbal drug use during pregnancy. This finding is comparable with a study done at University of Gondar teaching hospital which states that respondents who had monthly income lower than 100 USD were 3.1 times more likely to use herbal products than those who had an income greater than 200 USD [16]. Similarly, poor monthly income was reported to be associated with herbal drug use in a study done at public hospitals in Harar town of Ethiopia [18]. This is possibly explained by affordability of herbal medicine compared to modern medical services as indicated in other similar studies conducted in different parts of Ethiopia [5, 15, 16, 18] and Nigeria [27].

The main problems with herbal products are contamination, miss-labeling and absence of standardized dose in developing countries like Ethiopia [28, 29]. Similarly, participants of this study found information from neighbors, friends and family and got the herbal medicines from market or prepare it by themselves. This implies there is no dose standardization and controlled framework for manufacturing and distribution of herbal products. This may expose pregnant women and their fetuses to harmful effects.

### Limitation of the Study

Small sample size of this study may not reflect the average characteristics of pregnant women in the region. Thus, under-reporting of rarely used herbal medicines is expected. Additionally, this study is dependent on self-reported data which is subjected to bias.

## Conclusion

This study showed that half of the pregnant women attending ANC follow up at DRH used herbal medicine during their pregnancy. *Zingiber officinale, Allium sativum*, *Ocimum lamiifolium* and *Ruta chalepensis* were the herbs used. Being illiterate, having only primary and secondary school education background, and poor monthly income were the major factors contributing to herbal medicine use. Though there is excessive utilization of herbal medications during pregnancy, safety of the herbs (*Ocimum lamiifolium*) to the embryo or fetus is unknown indicating the need for future experimental studies to evaluate teratogenicity of herbal drugs in animal models. Health care providers, who are involved in antenatal care, should be aware of evidences regarding potential benefits and harmful effects of herbal products during pregnancy, and they should provide health education to pregnant women.

## Supplementary Information

**Additional file 1.** Questionnaire used for data collection

## Data Availability

The questionnaire for data collection is available as additional file. The raw data are available from the corresponding author upon reasonable request.

## Abbreviations

ANC: Antenatal Care
AOR: Adjusted odds ratio
CI: Confidence interval
COD: Crude odds ratio
DRH: Dessie referral hospital
