# Supplementary material for: A cross-sectional study of herbal medicine use and contributing factors among pregnant women on antenatal care follow-up at Dessie Referral Hospital, Northeast Ethiopia
Source: BMC Complement Med Ther. 2022 May 24;22:146. doi: 10.1186/s12906-022-03628-8 (PMC9131662; doi:10.1186/s12906-022-03628-8)
Supplement: Supplementary file 1 — Additional file 1. Questionnaire used for data collection [file 12906_2022_3628_MOESM1_ESM.docx]

**WOLLO UNIVERSITY**

**COLLEGE OF MEDICINE AND HEALTH SCIENCES**

**DEPARTMENT OF PHARMACY**

**Questionnaire used for data collection**

**Consent information sheet**

My name is ____________. I am here on behalf of the researchers from department of Pharmacy, Wollo University. We are conducting a research on ‘Herbal Medicine Use and Contributing Factors among Pregnant Women on Antenatal Care Follow-up at Dessie Referral Hospital, northeast Ethiopia’. The study has been approved by the ethical review committee of college of medicine and health sciences, Wollo University. The aim of this study is to determine the prevalence of herbal medicine use, identify the type of herbal medicines used and contributing factors for herbal medicine use. The information you provide has great value for the success of this study. It will eventually help for better understanding of the problem and for designing appropriate interventions for the problem. You are selected randomly to participate in this study. Your participation is purely based on your willingness. You have the right to choose not to take part in this study. If you choose to take part, you have the right to stop at any time. If you agree to participate in the study, the information that you provide will be kept confidential.

Based on the information I gave you, are you willing to participate in this study?

Yes No

**Thank you for your willingness to participate in the study!!**

**Name of data collector_______________________________**

**Signature__________________________________________**

**Date of data collection________________________________**

**Section A: Socio-Demographic Information**

1. Age (in years); _______________

2. Marital status;

a) single

b) married

c) divorced

d) widowed

3. Occupation;

a) Governmental employed

b) self-employee

c) Housewife

d) Farmer

e) Student

f) Other (specify) ________________

4. Monthly income (in Ethiopian birr) ______________________

5. Education level;

a) Illiterate

b) Primary school (1-8)

c) Secondary school (9-12)

d) College/University student

e) Diploma/Degree

f) Other (specify) ______

6. Ethnicity;

a) Oromo

b) Amhara

c) Tigre

d) Others _______

7. Religion;

a) Orthodox

b) Muslim

c) Protestant

d) Other (specify) ______

8. Place of Residence; a) Urban b) Rural

9. Distance from health facility (hospital or health center);

a) <5 km

b) 5-10 km

c) >10 km

**Section B: Obstetrics Information**

1. Gravida; ____________

2. Number of child (Parity); ___________

3. Previous abortion;

a. Yes

b. No

4. If yes, the reason for abortion (specify it) _________________

5. Stage of pregnancy;

a) First trimester

b) Second trimester

c) Third trimester

**Section C: Herbal Medicine Use**

1. Have you practiced herbal medicine during prior pregnancies?

a) Yes

b) No

2. Have you practiced herbal medicine during the current pregnancy?

a) Yes

b) No

3. If your answer for question number 2 is no, what is your reason?

1. Lack of belief in the benefits of herbs
2. Afraid the side effect
3. Lack of availability
4. Didn’t get sick during gestation
5. Other (specify)_____________

4. Reason for herbal medicine use?

a) Herbal medicines are effective than conventional medicines

b) Herbal medicines have fewer side effects

c) Herbal medicines have lower cost

d) Herbal medicines are accessible without prescription

e) Other (specify)_____________________

5. For what purpose and ailments did you use herbal medicine?

a) Headache

b) Nausea/Vomiting

c) Typhoid

d) UTI

e) Common cold

f) Diarrhea

g) To facilitate labor

h) To prevent abortion

i) Other (specify)______

6. What type of herb(s) have you used?

a) Ginger

b) Garlic

c) *Ruta chalepensis* (Tena-adam)

d) *Ocimum lamifolium* (Damakese)

e) Thyme (Tosign)

f) Other (specify)_____________

7. Who is your source of information about herbal medicine?

a) Traditional healers

b) Health professionals

c) Religious leaders

d) Family and friends

e) Neighbors

f) Other (specify)____________

8. Where did you get the herbal medicines you used?

a) Self-preparation

b) Traditional healers/Herbalist

c) Traditional birth attendants

d) Market place

e) Neighbors

f) Other (specify)___________

9. Have you had any unwanted effects from herbal medicines use? a) Yes b) No

10. In general, how could you rate the advantage you get from using herbal medicines use?

a) Satisfied

b) Average

c) Dissatisfied
